# Early intervention in mental health: The best bet

**DOI:** 10.1016/j.lansea.2022.100090

**Published:** 2022-10-03

**Authors:** The Lancet Regional Health – Southeast Asia

Mental disorders are the leading cause of disability and a major cause of death. In 2019, around 970 million people (one in eight) lived with a mental disorder worldwide, of whom close to 200 million lived in south Asia including almost a third of people who have depression worldwide. A recent *Lancet* Article revealed that from 2017 to 2018, close to 8% of people aged 45 years and older in India had mental health conditions. Most were undiagnosed, and the majority of those who had a diagnosis had no access to treatment or support.

In addition to low health budgets allotted to mental health care, there is a huge gap in affordability, accessibility, and poor quality of services. The WHO Comprehensive Mental Health Action Plan 2013–30 provides a blueprint for action on commitment to mental health care. Even though some countries in the region including Bangladesh, Indonesia, Sri Lanka, and India have instituted mental health policies to tackle the problem at a national scale, the region is still severely short on care and services leaving millions without treatment and support.

Treatment and care for mental health disorders backed by scientific evidence have been a challenge so far. Not only because of the complexity of disorders, but also because most people access care in the late stages of disease. As many mental disorders might have an onset before the age of 18 years, identifying risk factors and early treatment across the population becomes imperative for reducing the burden on health systems and quality of life.

A large proportion of the young population is bearing a substantial burden of mental health disorders. Identification of these disorders in the early stages and proper treatment can accord right to healthy and productive personal and work lives. The State of the World's Children 2021 report found that around one in seven people aged 15–24 years in India reported symptoms of depression. Less than half of these people felt comfortable to reach out for mental health support. Sadly, India records the highest number of suicide deaths in the world, with suicide being the leading cause of death in the 15–39 years age group. This fact aptly expresses the need to address low awareness and stigma around mental health issues at a younger age and develop strategies that include community and whole of society approaches.

Research reveals that community engagement is the key to building inclusive environments for mental health problems. Cultural complexity makes it essential to understand several social and cultural risk factors, and the reluctance to seek care in the region. Traditionally, spiritual practices and community festivals can have a positive impact on mental health. Stigma associated with mental health problems needs to be understood at the community level and strategies to address those factors need to be included in mental health programmes.

Attempts at engaging the community for mental health services in the region have included rehabilitation, life-skills training, and resilience building around social issues. Few organisations have started mobile applications for stress relief and helplines for women and children in distress. However, people living with disabilities and those in imprisonment, conflict areas, extreme poverty and destitute, and refugees have no access to such services. A transdisciplinary programme launched in India by the National Institute of Mental Health and Neurological Sciences for children and adolescents who have experienced abuse or adverse events in early life is an effective strategy that can been replicated at national level. In this issue of *The Lancet Regional Health Southeast Asia*, Khin Gyee and colleagues showed how upskilling of primary health-care workers in mental health and use of appropriate digital technology could improve integration of mental health services into primary care and reduce the treatment gap.

The severity of mental health issues is often neglected throughout the region leaving millions without care and support. With scarce resources and gaps in service delivery, an approach similar to that used in other chronic diseases needs to be recognised and should focus on prevention. Identification of risk factors, prevention, and early intervention targeting those risk factors could provide wider benefits in the long term. These services when integrated in the community are a message of hope for people with mental distress for the fear of discrimination and stigma. Investing in prevention, early treatment, and population-based approaches for future mental health and wellbeing are the best bet.

